# Effects of Complex Environmental Conditions on Fatigue Self-Healing Properties of Fast-Melting SBS-Modified Asphalt

**DOI:** 10.3390/ma18092157

**Published:** 2025-05-07

**Authors:** Jinchao Yue, Jiahao Fu, Yihan Wang, Yang Ming, Guoqi Tang, Ruixia Li

**Affiliations:** 1 School of Water Conservancy and Transportation, Zhengzhou University, Zhengzhou 450001, China; yuejc@zzu.edu.cn (J.Y.); fujh38542426@163.com (J.F.); 17513162919@163.com (Y.W.); 2 Henan Transport Investment Group Co., Ltd., Zhengzhou 450046, China; 13087025558@163.com; 3 Guolu Gaoke Engineering Technology Institute Co., Ltd., Beijing 100083, China; tang12341102@163.com

**Keywords:** fast-melting SBS, complex environmental, fatigue self-healing properties, modified asphalt

## Abstract

Asphalt pavements are prone to various distresses under complex environmental influences during service, which significantly affects their fatigue life. This study conducted complex environmental simulation tests, including pressure aging, ultraviolet (UV) aging, and coupling effects with salt solutions at different concentrations. A dynamic shear rheometer (DSR) was employed to perform frequency sweep tests, linear amplitude sweep (LAS) tests, and fatigue–healing–fatigue tests. The fatigue self-healing properties of fast-melting SBS (SBS-T)-modified asphalt were evaluated based on the viscoelastic continuous damage theory. The results indicate that coupled aging effects significantly increase the viscoelastic characteristic parameters of SBS-T-modified asphalt, with more elastic components transforming into viscous components. Compared to other aging effects, the coupled pressure-UV-salt solution condition induces the most severe degradation in the fatigue durability of SBS-T-modified asphalt. Simultaneously, the self-healing capability of aged asphalt is also reduced. Specifically, with increasing strain, more complex aging conditions lead to the faster deterioration of asphalt fatigue life and lower self-healing capacity. While asphalt demonstrates measurable fatigue life restoration through self-healing, the synergistic coupling of salt solution exposure and multi-factor aging significantly compromises both the absolute fatigue resistance and the relative recovery efficiency.

## 1. Introduction

Asphalt pavements are persistently subjected to vehicular loading in conjunction with environmental stressors, notably UV radiation, thermal oxidation, and moisture infiltration, during prolonged service periods, resulting in diverse distress manifestations such as fatigue cracking and thermal-induced fracturing. Asphalt aging is recognized as the primary cause of the reduced durability and shortened service life of high-grade highways. The prevention or mitigation of crack propagation to extend the service life of asphalt pavements has emerged as a critical research frontier in pavement sustainability engineering [1]. Among various pavement distresses, fatigue damage is particularly crucial to the service performance of asphalt pavements, as fatigue life directly determines the pavement’s service duration. Due to the unique characteristics of asphalt materials, their fatigue failure behavior is influenced by nonlinear viscoelastic effects, thixotropy, spatial hardening, and self-healing behavior [2]. Consequently, advancing the precision of asphalt fatigue life prediction necessitates the systematic incorporation of self-healing mechanisms into fatigue performance characterization frameworks.

The current research on the effects of external environments on the fatigue self-healing properties of asphalt materials primarily focuses on individual factors. However, in actual road service conditions, pavements are subjected to multiple simultaneous factors. Pressure, UV radiation, and saline environments can accelerate asphalt aging, significantly affecting its durability. Laboratory aging simulation tests, enabling precise parameter control and accelerated aging regimes, serve as standardized protocols to investigate asphalt aging mechanisms [3]. The Rolling Thin Film Oven Test (RTFOT) and Pressure Aging Vessel (PAV) are standardized protocols employed to mimic short-term oxidative aging and long-term thermomechanical degradation of asphalt binders, respectively. However, these accelerated aging protocols fall short of accurately reproducing in-service pavement environments owing to the exclusion of UV radiation and moisture-induced degradation mechanisms. Photooxidation is a major aging factor in regions with intense sunlight, particularly UV radiation from the solar spectrum, which significantly accelerates asphalt pavement aging [4]. Wang et al. established UV radiation as a dominant degradation mechanism in asphalt aging, inducing significant deterioration of fatigue resistance, elevation in creep stiffness, and reduction in failure strain [5]. Photooxidative pathways, when coupled with thermal oxidation kinetics, enable a more accurate replication of service-induced aging processes compared to conventional thermal aging protocols, positioning UV exposure as a critical parameter in comprehensive durability assessments [6].

In China’s inland and coastal saline–alkali regions, harsh natural conditions make asphalt pavements and bridge decks more susceptible to various early distresses. When exposed to rainwater, salt components in the soil can adversely affect asphalt pavements in solution form [7]. Moreover, the water contacting asphalt pavements is not pure, as salts used for snow and ice melting can form damaging solutions. Coastal regions, where roads and sidewalks are predominantly asphalt-paved, face additional challenges from atmospheric salt, rainwater, and sea fog containing substantial sea salt, which degrade the pavement performance and service life [8,9,10]. However, the current asphalt pavement material design in China still relies on conventional empirical methods, rarely considering the combined effects of UV radiation, pressure, and salt solutions on durability, making it inadequate for specific environmental demands. There is an urgent need to investigate the deterioration dynamics of asphalt under synergistic environmental stressors, which is critical for advancing pavement sustainability.

Asphalt, particularly polymer-modified asphalt, is the primary material for expressway construction in China, and its viscoelastic response in complex environments fundamentally governs the structural integrity of asphalt concrete pavements and their long-term serviceability [11,12]. The SBS modifier has emerged as the primary choice for asphalt modification owing to its superior compatibility with base asphalt coupled with the ability to impart exceptional elasticity and enhanced high-temperature performance to modified asphalt systems [13]. However, traditional wet process SBS-modified asphalt production faces technical and management challenges, including segregation and aging issues that are difficult to monitor and control, significantly affecting performance [14,15]. Considering these limitations, the dry process, where modifiers are directly mixed with base asphalt in the mixer, shows superior advantages by eliminating the modification production process. This approach not only prevents aging caused by pre-mixing asphalt with modifiers during production but also reduces carbon emissions compared to conventional modification methods, making it more environmentally friendly [16]. The widespread adoption of dry modification technology is not only an industry necessity but also provides crucial technical support for green road construction. Research [17] on the pavement performance of SBS-T modifiers showed that all technical requirements met the specification standards, with some indicators surpassing those of wet process asphalt. With increasing applications of dry technology in practical engineering, it is essential to focus on SBS-T-modified asphalt performance to provide reference data for engineering applications.

In summary, to thoroughly investigate the combined effects of pressure, UV radiation, and salt solutions on the fatigue self-healing properties of SBS-T-modified asphalt, the current investigation conducted experiments under designed conditions of coupled pressure, UV, and saline environments. Using dynamic shear rheological tests and based on the viscoelastic continuous damage theory, the research predicts asphalt fatigue life to reveal the influence patterns of complex environments on asphalt’s fatigue self-healing performance.

## 2. Materials and Methods

### 2.1. Materials

The base asphalt (70#:Penetration Grade 70, petroleum asphalt with a penetration range of 60–80 (0.1 mm) under standard test conditions at 25 °C) utilized in this investigation was procured from Beijing Guolu Gaoke Engineering Technology Institute Co., Ltd. The fundamental properties of the asphalt were tested in accordance with the “Standard Test Methods of Bitumen and Bituminous Mixtures for Highway Engineering”. The detailed test results are presented in Table 1.

The SBS-T modifier used for preparing polymer-modified asphalt was also provided by Beijing Guolu Gaoke Engineering Technology Institute Co., Ltd. The technical specifications of the modifier are detailed in Table 2.

### 2.2. Preparation of Fast-Melting SBS-Modified Asphalt

**Figure 1:**
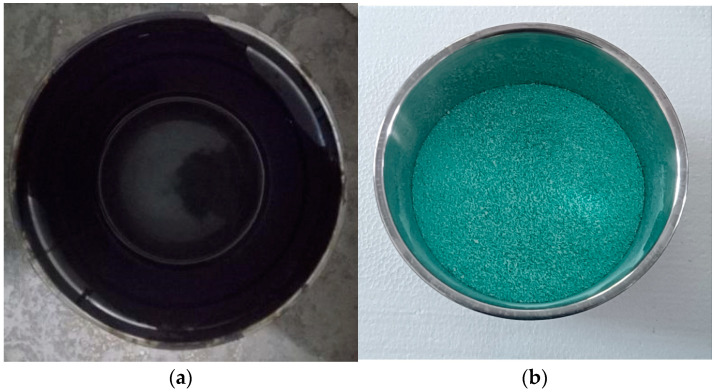
(**a**) Fast-melting SBS-modified asphalt and (**b**) SBS-T.

Previous studies have demonstrated that, for both radial and axial SBS-T-modified asphalt, when the modifier content exceeds 4%, it can meet all technical specifications required for construction-grade modified asphalt. Since the 4% dosage of radial SBS-T is commonly adopted in actual construction practices [19], this study utilized a 4% concentration of SBS-T modifier to prepare the modified asphalt, as illustrated in Figure 1. To align the properties of the laboratory-prepared asphalt with those of modified asphalt used in practical engineering, the following preparation protocol was adopted:

(1) The base asphalt was subjected to thermal treatment at 180 °C under continuous mechanical agitation, followed by incremental incorporation of the predetermined dosage of SBS-T modifier while maintaining a rotational speed of 800 rpm to ensure homogeneous dispersion.

(2) The complete addition of SBS-T was accomplished within 2 min, following the principle of multiple small additions to ensure uniform dispersion and prevent agglomeration. Intermittent sampling for fluorescence microscopy analysis was conducted to verify phase compatibility and swelling completeness, thereby facilitating the complete swelling of SBS-T.

(3) Following complete incorporation of the SBS-T modifier, the system was subjected to high-shear homogenization at 5000 rpm for 40 min under strictly controlled thermal conditions (180–190 °C). The temperature was continuously monitored throughout the process to ensure proper modification, resulting in the final SBS-T-modified asphalt product.

### 2.3. Preparation of Salt Solutions

The salt compound selected for simulating saline environments in this study was sodium chloride (NaCl). Considering the actual salt composition in field conditions, salt solutions with concentrations of 6%, 8%, and 10% were formulated using NaCl crystals. These distinct concentration gradients were designed to simulate the effects of varying deicing salt dosages on the performance characteristics of SBS-T-modified asphalt.

### 2.4. Aging Process Simulation Tests

#### 2.4.1. UV–Pressure Aging Test

To initiate the study, pour the SBS-T-modified asphalt sample evenly into a sample bottle with dimensions of 139.7 ±1.5 mm in height, 64 ±1.2 mm outer diameter, 2.4 ± 0.3 mm wall thickness, and 31.75 ±1.5 mm mouth diameter, containing 35 ± 0.5 g of asphalt. Place the sample bottle in a rotating thin-film oven maintained at a constant temperature of 163 ± 0.5 °C. The annular rack holding the sample bottles rotates at 15 ± 0.2 r/min while simultaneously injecting hot air at a flow rate of 4000 ± 200 mL/min. This process is maintained for 85 min. This procedure is designed to simulate the short-term aging process of asphalt. Following RTFOT conditioning to simulate short-term aging, the SBS-T-modified asphalt specimens were subjected to pressurized aging in a PAV for long term. Subsequently, the aged asphalt specimens were exposed to UV radiation in an ultraviolet aging chamber to replicate the synergistic interactions between thermal–oxidative degradation and UV-induced photoaging mechanisms.

**Figure 2:**
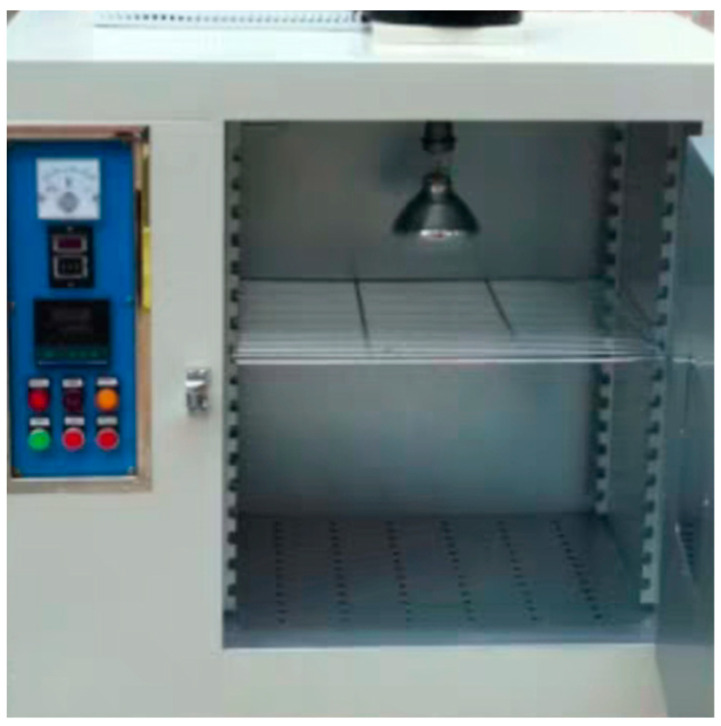
UV aging chamber.

From Figure 2, the ultraviolet aging system incorporated a 1.0 kW mercury–xenon arc lamp with spectral output spanning 345–355 nm. Samples were maintained at a 50 cm distance from the irradiation source, achieving a measured irradiance of 150 W/m^2^ via the calibrated UV radiometer. Temperature control ensured a regulated internal chamber environment of 45 ± 1 °C. Based on previous studies [20,21], most researchers adopt UV aging durations of less than 15 days. Therefore, a 7-day UV exposure period was selected for this study, consistent with the literature. This duration ensures significant aging acceleration while balancing experimental efficiency and avoiding under-degradation risks [22].

#### 2.4.2. UV–Salt Solution Aging Test

**Figure 3:**
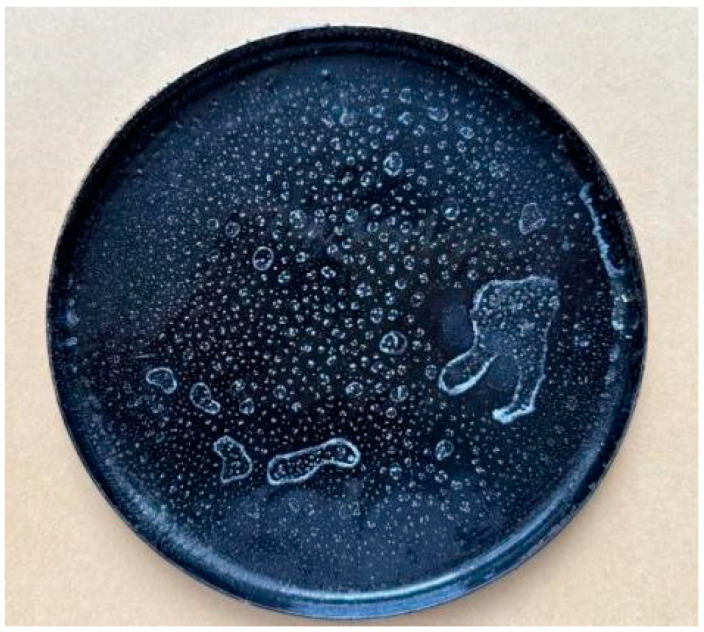
SBS-T-modified asphalt after UV–salt solution coupled aging.

The short-term aged asphalt was placed in an asphalt heating chamber and heated to a molten state (160 °C) to ensure sufficient fluidity for separation and weighing. Subsequently, 20 g of asphalt residue was weighed and poured into a tray to form a uniform thin film with a controlled thickness of ≤2 mm. These specimens were subsequently subjected to UV aging in the chamber using the procedure described in Section 2.4.1. To introduce saline environmental effects, the controlled daily spraying of prepared salt solutions at varying concentrations (6%, 8%, and 10%) was performed on the asphalt surface during UV exposure. Figure 3 illustrates the asphalt specimens after completing the coupled UV–salt solution aging process.

#### 2.4.3. UV–Pressure–Salt Solution Aging Test

A total of 50 g of short-term aged asphalt was placed in trays for long-term aging. Subsequently, 20 g of the long-term aged asphalt was weighed and placed in trays for UV aging. To avoid the influence of high temperatures on the salt solution, pre-configured salt solutions were sprayed daily onto the asphalt surface during the UV aging process. The pressure aging and UV aging procedures followed the same steps as described in the previous sections.

### 2.5. Experimental Protocols

#### 2.5.1. Frequency Sweep Test

Frequency sweep rheometry serves as a fundamental technique for rheological characterization of asphalt binders’ viscoelastic response. By applying varying loading frequencies and small strain levels at specific temperatures, the dynamic shear modulus of asphalt within its linear viscoelastic range can be determined.

A strain amplitude of 0.1% was applied within the linear viscoelastic regime of asphalt binders, which generally demonstrate linear behavior up to 15% strain. Rheological characterization was performed using a DSR under oscillatory loading frequencies spanning 0.2–30 Hz (12 discrete frequencies: 0.2, 0.4, 0.6, 0.8, 1, 2, 4, 6, 8, 10, 20, and 30 Hz) across three isothermal conditions: 15 °C, 25 °C, and 35 °C. Continuous monitoring captured the complex shear modulus (G*) and phase angle (*δ*) evolution [23,24].

Implementing the time–temperature superposition principle (TTSP) with 25 °C as the reference temperature, horizontal shifting factors were computationally derived to superimpose complex modulus data acquired at 15 °C, 20 °C, 30 °C, and 35 °C, thereby constructing a unified master curve characterizing the asphalt’s viscoelastic response across extended frequency–temperature domains. The shift factors were quantified under reference temperature conditions, and the reduced angular frequency used in the master curve is expressed as

(1) $$\log\;\omega_{r}=log\varphi_{T}+log\omega ,$$

where ω is the angular frequency (rad/s), $\omega_{r}$ is the reduced angular frequency (rad/s), and $\varphi_{T}\;is\;the\;shift\;factor$.

#### 2.5.2. Linear Amplitude Sweep (LAS) Test

**Figure 4:**
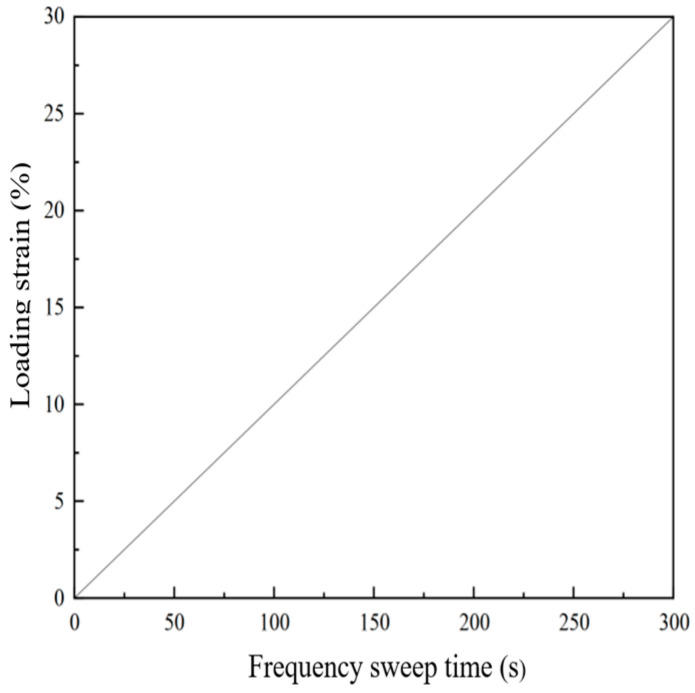
Loading protocol.

The LAS test, based on the Viscoelastic Continuum Damage (VECD) theory [25], employs a strain-controlled loading mode. Nonlinear fitting of the stress–strain data obtained from the LAS test allows the application of the VECD model, enabling the derivation of the damage characteristic curve (DCC) [26]. This curve provides a foundation for predicting fatigue performance. The loading protocol is illustrated in Figure 4. Continuous monitoring of dynamic modulus evolution, phase angle variation, and nonlinear viscoelastic response enables rigorous fatigue life prediction through the VECD theory framework.

The VECD theory is a theoretical model used to describe the behavior of materials under dynamic loading. This model integrates viscoelasticity and continuum damage mechanics, enabling the characterization of strength and stiffness reduction under high strain rates, as well as the fracture and damage evolution in materials. This methodology demonstrates significant applicability in fatigue characterization of asphalt materials, particularly for evaluating the resistance to crack propagation under cyclic loading conditions. The DCC of a material can be determined through simple monotonic loading strength tests. The DCC effectively describes the initiation and evolution of damage [27]. The damage evolution process is typically represented by Equations (2) and (3):

(2) $$W^{R}=f\left(\varepsilon^{R},S\right)=\frac{1}{2}\sigma \varepsilon^{R},$$

(3) $$\frac{\mathrm{dS}}{\mathrm{dt}}={\;\left(-\frac{\partial W^{R}}{\partial S}\right)}^{\alpha },$$

where $W^{R}$ is the strain energy parameter, $\varepsilon^{R}$ is the strain parameter, S is the internal damage variable (typically represented by modulus), σ is the stress parameter, and α = material constant in the undamaged state.

To apply the Viscoelastic Continuum Damage (VECD) theory to asphalt, Equation (4) is used in place of Equation (2):

(4) $$W=\pi {\cdot I}_{D}\cdot \gamma_{0}^{2}\cdot \left|G^{*}\right|\cdot \sin\delta ,$$

where $I_{D}$ is the dynamic shear modulus in the undamaged state, $\gamma_{0}$ is the strain amplitude (%), $\left|G^{*}\right|$ is the complex shear modulus, and δ is the phase angle.

The damage parameter D is quantified by linking the modulus and damage through the following equation:

(5) $$D\left(t\right)≅\sum_{i=1}^{N}{\left[\pi \gamma_{0}^{2}\left(\left|G^{*}\right|\sin\delta_{i-1}-\left|G^{*}\right|\sin\delta_{i}\right)\right]}^{\frac{\alpha }{1+\alpha }}{\left(t_{i}-t_{i}\right)}^{\frac{1}{1+\alpha }}$$

where $I_{D}$ is the dynamic shear modulus in the undamaged state, $\gamma_{0}$ is the strain amplitude (%), $\left|G^{*}\right|$ is the complex shear modulus, and δ is the phase angle.

The damage parameter *D* is quantified by linking the modulus and damage through the following equation:

(6) $$G^{\prime }=\left|G^{*}\right|\;\times \cos\delta ,$$

(7) $$G^{\prime \prime }=\left|G^{*}\right|\times \sin\delta ,$$

By taking the logarithm of the storage modulus G′ and angular frequency *ω*, the linear fit in Equation (8) yields the parameter *m*:

(8) $$\log G^{\prime }=m\left(\log\omega \right)+b,$$

The rheological parameter *α* is then calculated as

(9) $$\alpha =\frac{1}{m},$$

To establish the relationship between the internal state of the material and the damage parameter, the following fitting model is used:

(10) $$C\left(t\right)=C_{0}-C_{1}{\left[D\left(t\right)\right]}^{C_{2}},$$

where $C_{0}=1$, and the fitting parameters $C_{1}$ and $C_{2}$ are derived from

(11) $$\mathrm{lg}\left[C_{0}-C\left(t\right)\right]=\mathrm{lg}C_{1}+C_{2}\mathrm{lg}\left[D\left(t\right)\right],$$

The fatigue failure criterion evaluates asphalt fatigue damage and predicts fatigue life under varying loading conditions. According to AASHTO TP 101-12, fatigue failure is defined when the initial $\left|G^{*}\right|\sin\delta$ drops to 35% (i.e., C=0.65). However, this criterion treats all asphalt samples uniformly, ignoring material variability and leading to less accurate fatigue life predictions.

In contrast, AASHTOTP 101-14 uses the C value corresponding to the peak stress in the stress–strain curve as the fatigue failure point. Zhou et al. [28] validated both criteria, demonstrating that the peak stress-based C value better reflects true fatigue failure with lower variability. Therefore, this study adopts the peak stress-based C value for fatigue life calculation. The fatigue damage failure criterion ($D_{f}$) is expressed as

(12) $$D_{f}={\left(\frac{C_{0-}C_{\mathrm{peak}}}{C_{1}}\right)}^{\frac{1}{C2}},$$

where $C_{\mathrm{peak}}\;$is the C(t) value at peak stress.

The fatigue equation parameters A and B are calculated as

(13) $$A=\frac{f{\left(D_{f}\right)}^{k}}{k{\left(\pi C_{1}C_{2}\right)}^{\alpha }},B=2\alpha ,$$

The final fatigue life equation, used to predict asphalt fatigue life at any strain level, is

(14) $$N=A{\left(\gamma_{\max}\right)}^{-B},$$

where f *=* 10 Hz, and $k=1+\left(1-C_{2}\right)\alpha$.

#### 2.5.3. Fatigue–Healing–Fatigue Test

Xie et al. [29] proposed the linear amplitude sweep-based healing test (LASH) to quantify the self-healing properties of asphalt. In this study, the LASH test was conducted by introducing a rest period into the standard LAS test. The self-healing performance was evaluated by comparing the damage variable D before and after the rest period.

The key aspects of the LASH test include the selection of the damage level and the healing time. For the asphalt samples subjected to coupled aging in this study, the LAS test was first performed to determine the damage parameter $D_{f}$ at fatigue failure, defined by the peak stress. A damage level of 50% $D_{f}$ was selected as the threshold for initiating the healing process, and the corresponding strain level was calculated.

**Figure 5:**
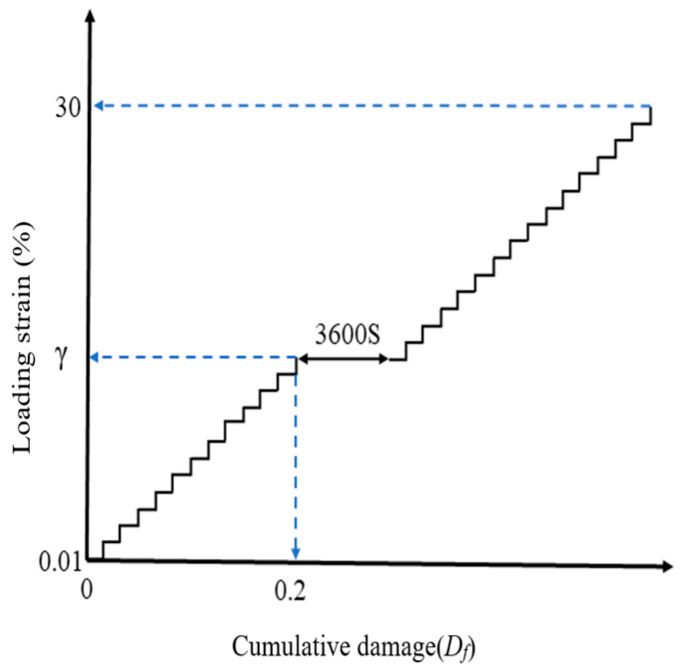
Linear amplitude sweep-based healing test protocol.

A new asphalt specimen was then subjected to the LAS test at the standard loading rate. When the strain reached the predetermined level (corresponding to 50% $D_{f}$), the loading was stopped, and the specimen was allowed to rest for one hour at a constant temperature of 25 °C. The second loading phase began with the same strain amplitude and loading rate as the end of the first phase. The loading protocol is illustrated in Figure 5.

## 3. Results and Discussion

### 3.1. Linear Viscoelastic Analysis (Continued)

**Figure 6:**
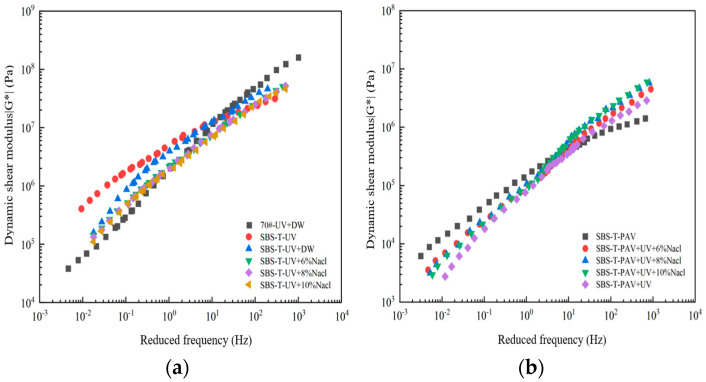
(**a**) UV–salt solution aging, and (**b**) UV–pressure aging and UV–pressure–salt solution aging.

The dynamic shear modulus ($G^{*}$) is a critical parameter for characterizing the viscous and elastic behavior of asphalt materials under dynamic loading. An increase in $G^{*}$ at low frequencies (high temperatures) enhances the asphalt’s resistance to high-temperature deformation. Conversely, an increase in $G^{*}$ at high frequencies (low temperatures) leads to hardening and embrittlement, resulting in poor low-temperature performance. The master curves of the dynamic shear modulus for SBS-T-modified asphalt under different coupled aging conditions are shown in Figure 6.

**Figure 7:**
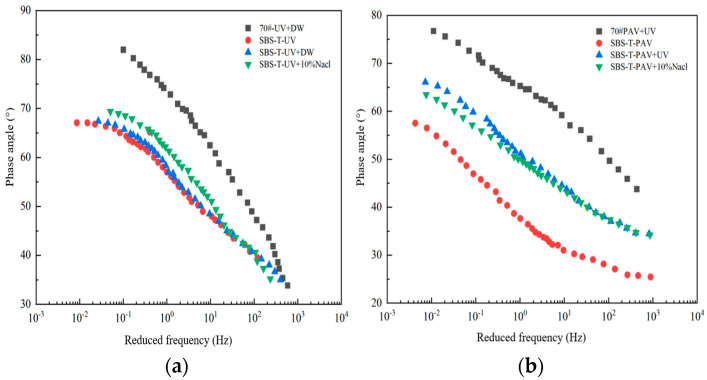
(**a**) UVsalt solution aging, and (**b**) UVpressure aging and UVpressuresalt solution aging.

As shown in Figure 7, in the low-frequency (high-temperature) region, SBS-T-modified asphalt exhibits a higher dynamic shear modulus ($G^{*}$) compared to base asphalt. In contrast, in the high-frequency (low-temperature) region, the base asphalt demonstrates a higher $G^{*}$ than SBS-T-modified asphalt. The significant enhancement in modulus across the entire range indicates that base asphalt is more sensitive to temperature variations, leading to poor performance at both low and high temperatures. Specifically, it is prone to cracking at low temperatures and deformation at high temperatures. On the other hand, SBS-T-modified asphalt exhibits superior high-temperature deformation resistance and low-temperature crack resistance.

In the low-frequency region (Figure 6a), the dynamic shear modulus of SBS-T-modified asphalt under different aging conditions follows the order of UV aging alone > UV–deionized water coupling > UV–salt solution coupling. The UV–salt solution coupling significantly reduces $G^{*}$ compared to UV aging alone, indicating that the combined effect of UV and salt solution deteriorates the deformation resistance of asphalt. In contrast, as shown in Figure 6b, the dynamic modulus of SBS-T-modified asphalt under UV–pressure–salt solution coupling is higher than that under UV–pressure coupling, suggesting that the addition of salt solution improves the deformation resistance when combined with pressure and UV aging.

In the high-frequency region, SBS-T-modified asphalt subjected to UV aging alone exhibits a lower $G^{*}$, while UV–deionized water and UV–salt solution coupling result in higher $G^{*}$. This indicates that coupling UV aging with these factors hardens and embrittles the asphalt, reducing its low-temperature crack resistance. However, the dynamic modulus of asphalt decreases after UV–salt solution aging, suggesting that the salt solution improves the low-temperature performance of SBS. Conversely, the dynamic modulus increases after UV–pressure–salt solution aging, indicating that the salt solution weakens the crack resistance under these conditions.

The phase angle (δ) represents the ratio of viscous-to-elastic response in asphalt and reflects the material’s viscoelastic behavior. A higher δ indicates a greater viscous component, leading to poorer deformation resistance at high temperatures. Conversely, a lower *δ* suggests a stronger elastic response, enhancing the material’s ability to resist deformation. The master curves of δ for asphalt under different aging conditions are shown in Figure 7.

As shown in Figure 7, the phase angle (δ) master curves of all asphalt samples generally decrease with increasing frequency. This trend occurs because, as the frequency increases, the loading duration shortens, causing the asphalt’s deformation to transition from viscous to elastic behavior, thereby reducing the phase angle. Under the same aging conditions, the phase angle curves of base asphalt are consistently above those of SBS-T-modified asphalt, indicating that SBS-T modification enhances the deformation resistance of asphalt.

As aging factors become more complex, the phase angle increases, suggesting that coupled aging introduces more viscous components into the asphalt, reducing its deformation resistance. Specifically, compared to UV aging alone, the UV–salt solution coupling results in a larger phase angle for SBS-T-modified asphalt, indicating that the salt solution accelerates aging and further hardens the asphalt. This hardening increases the viscous behavior of asphalt at high temperatures. The underlying mechanism may involve the intense UV aging reaction, where the introduction of a salt environment disrupts the colloidal structure balance within the asphalt, accelerating the breakdown of chemical bonds and their reaction with oxygen.

In contrast, when pressure aging is included in the coupling, the phase angle decreases significantly, indicating an increase in elastic components and enhanced deformation resistance. Compared to UV–pressure coupling, the UV–pressure–salt solution coupling results in a smaller phase angle, suggesting that the salt environment promotes a higher elastic response during pressure aging, further improving the asphalt’s deformation resistance.

### 3.2. Fatigue Performance Analysis

**Figure 8:**
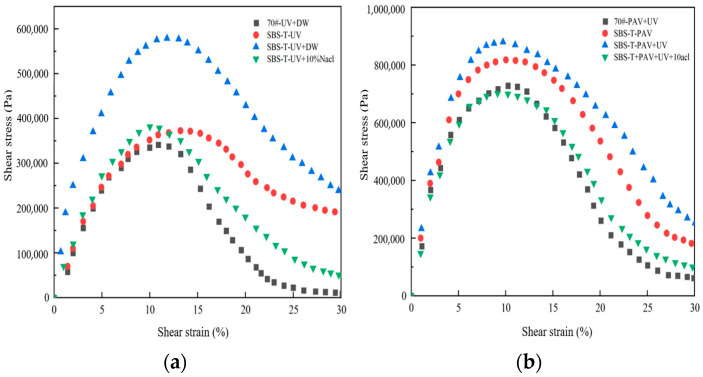
(**a**) UVsalt solution aging, and (**b**) UVpressure aging and UVpressuresalt solution aging.

The peak stress in the stress–strain curve was used as the fatigue failure criterion in the LAS test. Based on the VECD fatigue criterion, the stress–strain curves of asphalt under different aging conditions at 25 °C were analyzed, as shown in Figure 8.

From Figure 8, it can be observed that the peak stress of the asphalt material can be determined by analyzing the stress–strain curves, providing insights into the strain-dependent performance of the material. Under different coupled aging conditions, the asphalt exhibits peak stress, followed by a decrease in stress after the peak strain, indicating the occurrence of fatigue damage.

All asphalt samples display a plateau region at the peak stress, with a wider plateau indicating stronger resistance to loading. Under the same aging conditions, SBS-T-modified asphalt exhibits a broader peak stress region compared to base asphalt, and a plateau region emerges as the strain increases. This suggests that SBS-T-modified asphalt is less sensitive to strain and has enhanced deformation resistance, reflecting the influence of the SBS-T modifier on its viscoelastic properties. In the peak stress region, a decrease in stress signifies the initiation of fatigue damage. Compared to base asphalt, SBS-T-modified asphalt shows a slower stress reduction, indicating that strain variations have a lesser impact on stress.

As aging factors become more complex, the peak stress of SBS-T-modified asphalt increases, while the peak strain decreases. The peak stress of SBS-T-modified asphalt after UV–deionized water coupling is higher than that after UV–salt solution coupling. Similarly, the peak stress after UV–pressure coupling is greater than that after UV–pressure–salt solution coupling. The introduction of a salt environment significantly reduces the peak stress, indicating that the salt solution diminishes the deformation resistance and increases the strain dependency of the asphalt, adversely affecting the durability of asphalt pavements. Compared to pressure aging alone, UV–pressure coupling increases the peak stress of SBS-T-modified asphalt, suggesting that UV aging influences the deformation resistance, making the asphalt more susceptible to physicochemical changes under UV exposure.

**Figure 9:**
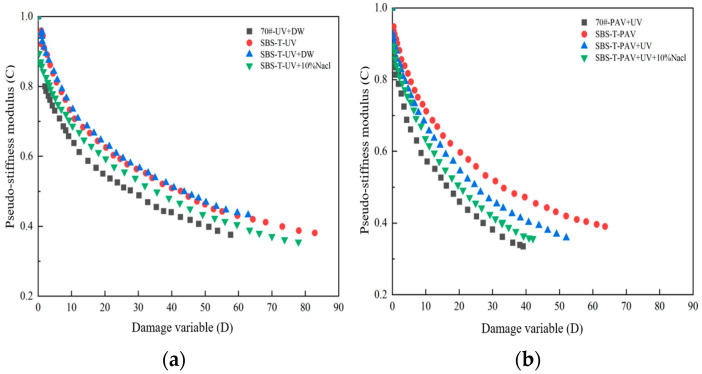
(**a**) UVsalt solution aging, and (**b**) UVpressure aging and UVpressuresalt solution aging.

The DCC, derived from the VECD theory and the peak stress fatigue criterion, reflects the relationship between pseudo-stiffness modulus (*C*) and cumulative damage variable (*D*). The DCC curves of aged asphalt are shown in Figure 9.

**Figure 10:**
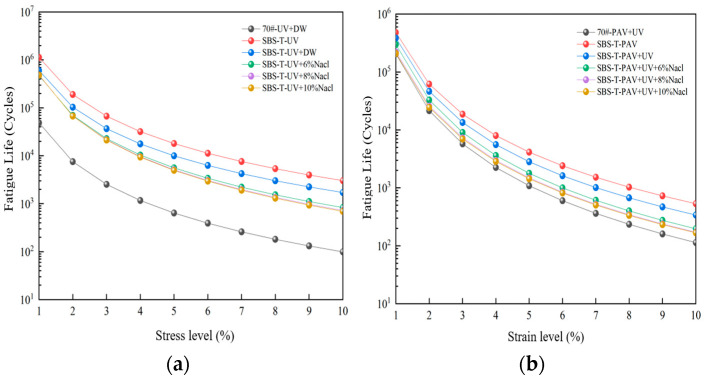
(**a**) UV–salt solution aging, and (**b**) UV–pressure aging and UV–pressure–salt solution aging.

Each DCC exhibits distinct *C_f_* and *D_f_* values, where a higher *D_f_* typically indicates better material durability. The endpoints of the curves in Figure 9, determined based on the peak stress fatigue criterion, represent the fundamental fatigue characteristics of the material. As shown in Figure 10, the endpoints of the DCC curves vary under different aging conditions, reflecting changes in the physical and chemical structures of the asphalt due to aging.

As loading continues, damage accumulates in the asphalt, and the pseudo-stiffness modulus (*C*) decreases from *C* = 1 to the defined fatigue damage point. The decreasing rate of the curve slows over time, indicating that the sensitivity of asphalt fatigue damage to loading gradually diminishes. Under the same aging conditions, base asphalt exhibits the smallest damage threshold, while SBS-T-modified asphalt shows higher damage thresholds, demonstrating that SBS-T modification enhances the fatigue durability of asphalt.

After coupled aging, the endpoints of the DCC curves decrease to varying degrees. The DCC curve of SBS-T-modified asphalt under UV–salt solution coupling lies below that of UV aging alone, indicating faster damage accumulation. This suggests that, at the same damage level, asphalt not exposed to a salt environment maintains better structural integrity, while the salt environment reduces fatigue performance.

Analyzing the four asphalt samples subjected to pressure aging, the DCC curves are ranked as follows: SBS-T–PAV > SBS-T–PAV + UV > SBS-T–PAV + UV + 10% NaCl > base asphalt–PAV + UV. This ranking demonstrates that, as aging factors become more complex, the performance degradation of SBS-T-modified asphalt becomes more severe, with the most significant deterioration occurring under the three-factor coupling (pressure, UV, and salt solution).

Using Equation (14), the fatigue life of asphalt at any strain level can be predicted. Figure 10 shows the predicted fatigue life of asphalt under different aging conditions at strain levels of 1–10%.

As illustrated in Figure 10, asphalt fatigue life exhibits an inverse correlation with the strain magnitude, declining progressively as the strain increases. The fatigue life of SBS-T-modified asphalt subjected to UV–salt solution coupled aging is notably shorter than that under UV aging alone, highlighting the detrimental synergistic effects of saline environments on fatigue resistance. This observation aligns with field evidence showing heightened susceptibility to fatigue cracking in salt-exposed pavements under sustained traffic loads.

While SBS-T-modified asphalt demonstrates superior fatigue life compared to base asphalt after UV–salt solution coupling, the inclusion of pressure aging diminishes this advantage. Specifically, pressure aging disrupts the colloidal structure of SBS-T-modified asphalt, reducing cohesion and accelerating crack initiation. Post-pressure–UV coupling, the fatigue life of SBS-T-modified asphalt further decreases, underscoring UV aging’s incremental degradation effect. The most severe fatigue life reduction (lowest among all the conditions) occurs under triaxial coupling (UV–pressure–salt), emphasizing the compounding influence of multi-factor aging.

**Figure 11:**
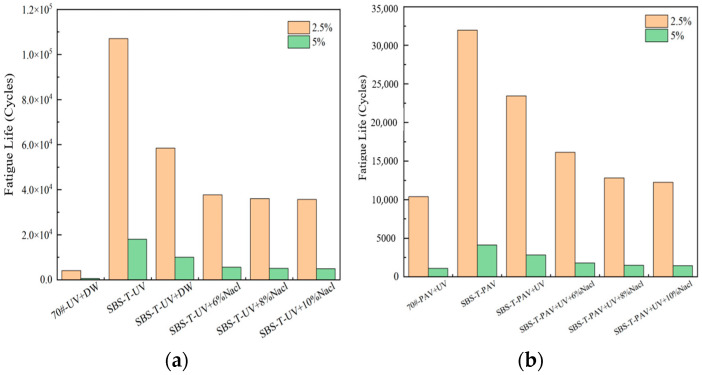
(**a**) UV–salt solution aging, and (**b**) UV–pressure aging and UV–pressure–salt solution aging.

Existing studies correlate the maximum strain thresholds ($\gamma_{max}$) with pavement layer thickness: $\gamma_{max}=2.5\%$ for thick layers and 5% for thin layers [30]. At$\gamma_{max}=5\%$, asphalt binder fatigue life ${(N}_{f})\;$strongly correlates with mixture-level fatigue performance [31]. This study calculated $N_{f}$ for two strain levels ($\gamma_{max}=2.5\%\;and\;\gamma_{max}=5\%$) across aging conditions, with the results summarized in Figure 11.

As illustrated in Figure 11, the addition of the SBS-T modifier enhances the fatigue life of asphalt. At both the 2.5% and 5% strain levels, the trends in fatigue life under different aging conditions are consistent. The fatigue life of SBS-T-modified asphalt after UV–salt solution coupling is shorter than that after UV aging alone, indicating that both UV radiation and the salt environment negatively impact the service life of asphalt pavements. When UV and salt solution coupling is applied, increasing the salt concentration has a minimal effect on fatigue life, suggesting that salt concentration plays a minor role in influencing fatigue performance under these conditions.

The fatigue life of SBS-T-modified asphalt after pressure–UV–salt solution coupling is the shortest among all the coupled aging conditions. This demonstrates that the combined action of these three factors disrupts the internal colloidal structure of the asphalt, destabilizing its matrix and reducing its modulus, thereby significantly impairing fatigue performance. In real-world pavement applications, the pressure–UV–salt solution coupling makes the pavement more prone to cracking, severely compromising its service life.

At the two predefined strain levels (2.5% and 5%), the fatigue life of SBS-T-modified asphalt is comparable to that of the base asphalt. At the lower strain level (2.5%), complex aging factors reduce the fatigue resistance of asphalt, and the fatigue life decreases to varying degrees under salt exposure. At the higher strain level (5%), SBS-T-modified asphalt demonstrates some advantages in fatigue resistance, but its resistance to salt-induced degradation remains insufficient.

### 3.3. Self-Healing Performance Analysis

In this study, the autogenous healing efficiency was quantified through Equation (15) to evaluate asphalt’s self-restorative capacity during intermittent loading cycles:

(15) $$\%H_{S}=\frac{D_{1}-D_{2}}{D_{1}}\times 100\%,$$

where $D_{1}$ is the damage variable before the rest period, and $D_{2}$ is the damage variable after the rest period.

**Figure 12:**
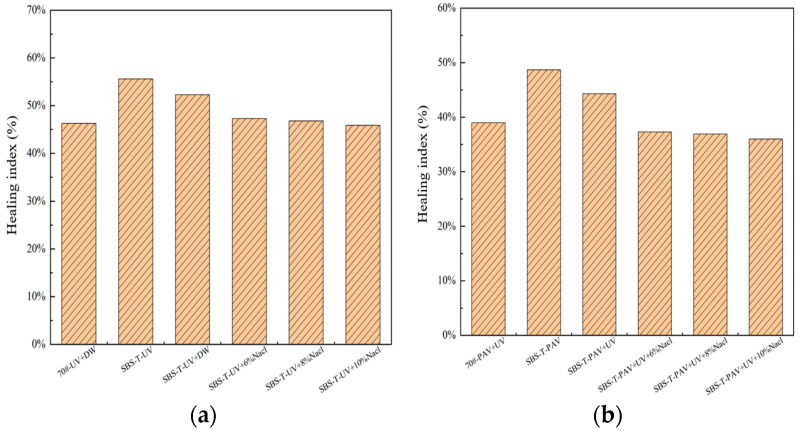
(**a**) UV–salt solution aging, and (**b**) UV–pressure aging and UV–pressure–salt solution aging.

The self-healing index, calculated using Equation (15), reflects the relationship between pseudo-stiffness and damage intensity. Elevated healing indices directly correlate with the enhanced intrinsic healing potential of the asphalt binder. Figure 12 presents a comparative evaluation of autogenous recovery efficiencies across specimens subjected to thermal–oxidative aging, UV-induced degradation, and their synergistic effects.

As illustrated in Figure 12, under the same aging conditions, SBS-T-modified asphalt exhibits a higher healing index ($\%H_{S}$) compared to base asphalt, indicating that the addition of the SBS-T modifier enhances the self-healing capability of asphalt. This improvement is attributed to the elastic recovery properties of the SBS-T modifier’s molecular chains.

Compared to UV aging alone, the healing index of SBS-T-modified asphalt significantly decreases after UV–salt solution coupling, demonstrating that the combined effect of these two aging factors reduces the healing capability of asphalt. When long-term aging factors are introduced, the self-healing efficiency of asphalt under pressure–UV–salt solution coupling is the lowest. This is largely due to UV-induced hardening, which degrades the mechanical properties of asphalt and hinders its healing process. Additionally, long-term aging causes significant changes in the chemical structure of asphalt molecules, slowing down their diffusion rate. These findings suggest that more complex aging environments lead to lower self-healing efficiency in SBS-T-modified asphalt.

Furthermore, analyzing the presence of salt solution in the aging conditions reveals that the healing index of SBS-T-modified asphalt decreases more significantly under salt solution coupling compared to deionized water exposure. This indicates that deionized water has a minimal impact on the healing behavior of asphalt, while salt solutions significantly deteriorate its self-healing capability. The deterioration is attributed to the adsorption of salt ions at the interface between asphalt molecules and the salt solution, which hinders the diffusion of asphalt molecules at the healing interface. Therefore, in real-world applications, particularly in coastal areas or during winter when deicing salts are used, the combined effects of UV radiation and long-term aging further reduce the self-healing ability and durability of asphalt pavements.

**Figure 13:**
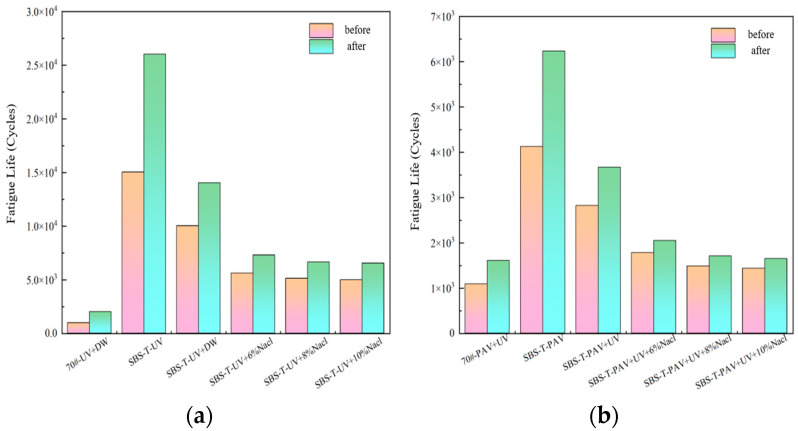
(**a**) UV–salt solution aging, and (**b**) UV–pressure aging and UV–pressure–salt solution aging.

The inclusion of a rest period allows partial recovery of the asphalt’s modulus, leading to an improvement in fatigue life. Based on the VECD theory, the fatigue life of asphalt before and after self-healing at a shear strain of 5% was calculated, as illustrated in Figure 13.

As illustrated in Figure 13, the fatigue life of asphalt increases significantly after the rest period. SBS-T-modified asphalt exhibits the most pronounced improvement in fatigue life after UV aging and pressure aging, while the growth rate of fatigue life decreases under multi-factor coupling conditions. This indicates that the combined effects of multiple aging factors hinder the recovery of the dynamic modulus during the rest period. Among the three coupled aging conditions, the inclusion of pressure aging has the greatest impact on the improvement of fatigue life before and after healing. This is attributed to the increased formation of hard components during long-term aging, which impedes the healing of internal microcracks and reduces the asphalt’s ability to withstand secondary loading.

Under aging conditions coupled with a salt environment, SBS-T-modified asphalt shows the lowest fatigue life and the smallest increase in fatigue life before and after healing. This demonstrates that salt solution erosion has a significant negative impact on the fatigue life of asphalt. Compared to previous analyses, when self-healing behavior is considered, all coupled aging conditions reduce the fatigue life of asphalt, with the most severe deterioration observed under UV–pressure–salt solution coupling.

## 4. Conclusions

This study investigated the effects of coupled aging factors—UV–salt solution, UV–pressure, and UV–pressure–salt solution—on the fatigue damage evolution and self-healing performance of asphalt using a dynamic shear rheometer and the Viscoelastic Continuum Damage (VECD) theory. The fatigue life of asphalt under different aging conditions was quantified, and the impact of coupled aging on self-healing performance was analyzed by evaluating healing indices and fatigue life changes. The main conclusions are as follows:

(1) The salt environment under UV–salt solution coupling makes SBS-T-modified asphalt more prone to deformation, reducing its high-temperature deformation resistance but improving its low-temperature crack resistance. Under UV–pressure–salt solution coupling, the participation of pressure aging enhances the high-temperature performance of asphalt but negatively affects its low-temperature performance.

(2) As aging factors become more complex, the peak stress of SBS-T-modified asphalt increases, while the peak strain decreases. The inclusion of pressure aging significantly accelerates the rate of peak stress reduction. Both UV–salt solution and UV–pressure–salt solution coupling reduce the peak stress of SBS-T-modified asphalt, indicating that the salt environment deteriorates the deformation resistance of asphalt.

(3) The pseudo-stiffness modulus of SBS-T-modified asphalt after UV–salt solution and UV–pressure–salt solution coupling is lower than that under single-factor aging, demonstrating that coupled aging reduces the deformation resistance. Under conditions involving pressure aging, the damage characteristic curve (DCC) shows a faster decline, indicating that pressure aging accelerates fatigue and weakens fatigue durability. The fatigue life of asphalt significantly decreases after coupling with salt solution and other aging factors, highlighting the adverse impact of salt solution on fatigue performance.

(4) Based on the VECD model and healing index, the self-healing performance of SBS-T-modified asphalt decreases after all three coupled aging conditions. The most significant reduction in self-healing index occurs under UV–pressure–salt solution coupling, where the self-healing index decreased by approximately 11% compared to UV–pressure aging alone. Furthermore, the post-healing fatigue life under this coupled aging condition was the lowest, showing a 70% reduction relative to UV–pressure aging. These results demonstrate that the combined effect of these three factors has the most pronounced impact on the self-healing performance of asphalt.

## Figures and Tables

**Table 1:** Basic properties of the base asphalt [18].

Property	Value
Ductility (25 °C, cm)	>100
Penetration (25 °C, 0.1 mm)	72
Dynamic viscosity (135 °C, Pa·s)	0.68
Softening point (°C)	50.5

**Table 2:** Technical indices of the SBS-T.

Indexes	Results
Mass of a single particle (g)	0.25
Styrene percentage (%)	30~32
Ash (%)	0.25
Dispersion of dry mixing	No particle residue

## Data Availability

The original contributions presented in this study are included in the article. Further inquiries can be directed to the corresponding author(s).
